# Effect of extended field‐of‐view approaches on the accuracy of stopping power ratio estimation for single‐energy computed tomography simulators

**DOI:** 10.1002/acm2.14010

**Published:** 2023-05-11

**Authors:** Chang‐Shiun Lin, Yi‐Chun Tsai, Liang‐Hsin Chen, Chun‐Wei Wang, Chia‐Jung Wu, Wan‐Yu Chen, Hsiang‐Kung Liang, Sung‐Hsin Kuo

**Affiliations:** ^1^ Department of Radiation Oncology National Taiwan University Cancer Center Taipei Taiwan; ^2^ Division of radiation oncology department of Oncology National Taiwan University Hospital Taipei Taiwan

**Keywords:** conversion curve, CT, extended field‐of‐view, Hounsfield unit, proton, stopping power ratio

## Abstract

**Background:**

Extended field‐of‐view (eFOV) methods have been proposed to generate larger demonstration FOVs for computed tomography (CT) simulators with a limited scanning FOV (sFOV) size in order to ensure accurate dose calculation and patient collision avoidance. Although the efficacy of these strategies has been evaluated for photon applications, the effect of stopping power ratio (SPR) estimation on proton therapy has not been studied. This study investigated the effect of an eFOV approach on the accuracy of SPR to water estimation in homogeneous and heterogeneous phantoms.

**Materials and Methods:**

To simulate patient geometries, tissue‐equivalent material (TEM) and customized extension phantoms were used. The TEM phantom supported various rod arrangements through predefined holes. Images were reconstructed to three FOV sizes using a commercial eFOV technique. A single‐energy CT stoichiometric method was used to generate Hounsfield unit (HU) to SPR (HU‐to‐SPR) conversion curves for each FOV. To investigate the effect of rod location in the sFOV and eFOV regions, eight TEM rods were placed at off‐center distances in the homogeneous phantom and scanned individually. Similarly, 16 TEM rods were placed in the heterogeneous TEM phantom and scanned simultaneously.

**Results:**

The conversion curves derived from the sFOV and eFOV data were identical. The average SPR differences of soft‐tissue, bone, and lung materials for rods placed at various off‐center locations were 3.3%, 4.8%, and 39.6%, respectively. In the heterogeneous phantom, the difference was within 1.0% in the absence of extension. However, in the presence of extension, the difference increased to 2.8% for all rods, except for lung materials, whose difference was 4.8%.

**Conclusions:**

When an eFOV method is used, the SPR variation in phantoms considerably increases for all TEM rods, especially for lung TEM rods. This phenomenon may substantially increase the uncertainty of HU‐to‐SPR conversion. Therefore, image reconstruction with a standard FOV size is recommended.

## 1 INTRODUCTION

Acquiring image sets from computed tomography (CT) simulators is a crucial initial step in radiotherapy. These images are used before treatment to delineate the target and calculate the dose. These CT images are required for the derivation of the relative electron density (RED) and stopping power ratio (SPR) to water. Therefore, the quantitation accuracy and image quality of these images are crucial for achieving precise and safe radiotherapy.^1^, ^2^, ^3^

To acquire images of the patient's body with various positioning and immobilization tools for CT simulation, a large bore size and scanning field‐of‐view (sFOV) are required. Insufficient data may result in calculation errors and increase the likelihood of patient collisions. Currently, the majority of CT simulators support sFOVs with a diameter of 50 cm^1^, ^4^, ^5^ while some manufacturers supporting larger sFOVs.^1^, ^6^, ^7^, ^8^ In other words, inadequate sFOV is common in radiotherapy applications. Therefore, truncated images are commonly obtained, particularly for obese patients with nonisocentric positioning. To account for missing data during image acquisition, several extended FOV (eFOV) techniques have been proposed^9^, ^10^, ^11^, ^12^ to obtain larger demonstration FOVs (dFOVs). These techniques aim to restore truncated information and eliminate streaking artifacts around the sFOV boundary while preserving the noise level and computational cost. In previous studies, the effects of eFOV techniques on image distortion, Hounsfield unit (HU) accuracy, and dosimetry have been investigated for photon beams.^4^, ^13^, ^14^ In the sFOV region, only limited HU deviation and image distortion were observed. However, in the extended region, large deviations were observed. In comparison to image sets reconstructed with a standard FOV (size of dFOV is identical to sFOV), the dose deviation in phantom studies was generally within 3%. Therefore, eFOV techniques are applicable in radiotherapy with high‐energy photons.

Recently, intensity‐modulated proton therapy (IMPT) has gained popularity worldwide because of its rapid dose fall‐off pattern at the distal end, conformal dose distribution, and low integral dose. However, the dose calculation accuracy of charged particles is extremely susceptible to uncertainties.^3^ One of the major uncertainties is the conversion of an HU (CT number) to a relative SPR to water. Depending on the tissue composition, the SPR uncertainty ranges from 1.6% to 5.0%.^15^ Currently, a margin design value of 3.5% is used at typical treatment sites. To avoid additional uncertainties, image reconstruction with sFOVs is used at the expense of a smaller dFOV. However, the demand for a wider FOV has continued to increase in this field. Generally, eFOV approaches are regarded as a potential alternative for CT simulators with a limited sFOV. Multiple studies have also addressed how to robustly obtain SPR values,^16^, ^17^, ^18^ to determine the effects of SPR estimation on dosimetry,^19^ and determine the effects of beam‐hardening correction (BHC) on SPR estimation.^5^ However, the influence of eFOV approaches on SPR estimation has not yet been evaluated. Therefore, this study investigated the feasibility of using eFOV techniques in proton therapy. A tissue‐equivalent material (TEM) phantom and a customized polymethyl methacrylate (PMMA) phantom were used to simulate homogeneous and heterogeneous configurations with a large patient geometry. In addition, the effects of eFOV approaches on HU‐to‐SPR conversion curves, the SPR variations of selected TEM rods placed in a homogeneous phantom in the eFOV region, and the SPR variations of a heterogeneous phantom were investigated.

## 2 MATERIALS AND METHODS

To determine the effects of eFOV approaches on proton therapy, SPR variations were evaluated with various phantom and reconstruction setups. A CT scanner with an sFOV diameter of 50 cm was installed at the study center. The scanner was used to acquire data in order to establish an HU‐to‐SPR conversion curve and conduct subsequent analyses. SPR evaluation was performed using an Eclipse commercial treatment planning system (version 16.0; VMS, Palo Alto, CA, USA) and DICOM tools in MATLAB R2021a (MathWorks, Natick, MA, USA). The CT scanner, reconstruction parameters, HU‐to‐SPR conversion curve generation, and phantom studies are described in detail in this section.

### 2.1 CT scanner and image acquisition

All measurements and reconstruction were conducted using a Siemens SOMATOM Definition Edge CT simulator (Siemens Healthineers, Erlangen, Germany). The bore size and diameter of the sFOV were set to 78 and 50 cm, respectively. This system supported both traditional eFOV and high definition (HD) FOV approaches. Different from a traditional eFOV image reconstruction, the HD FOV approach was used to ensure that the mean CT number and phantom diameter remain within 20 HU and 2 mm, respectively, in an electron density phantom (Tissue Characterization Phantom Model 467; Sun Nuclear, Melbourne, FL, USA).^20^ Phantoms were then scanned using an adult abdominal protocol (120 kVp, 128 × 0.6 mm, 3 mm slice thickness, 260 effective mAs) and reconstructed using the sinogram affirmed iterative reconstruction (SAFAIR) algorithm (strength = 1) with a default Qr40 kernel and a matrix size of 512 × 512. However, BHC was not used for complexity reduction. To determine the effect of the HD FOV approach (version Som/7 VB20) on the HU‐to‐SPR conversion curve and SPR estimation, the acquired data were reconstructed to FOVs with diameters of 50, 60, and 70 cm, with the FOV diameter of 50 cm used as the reference.

### 2.2 HU‐to‐SPR conversion curve

**FIGURE 1:**
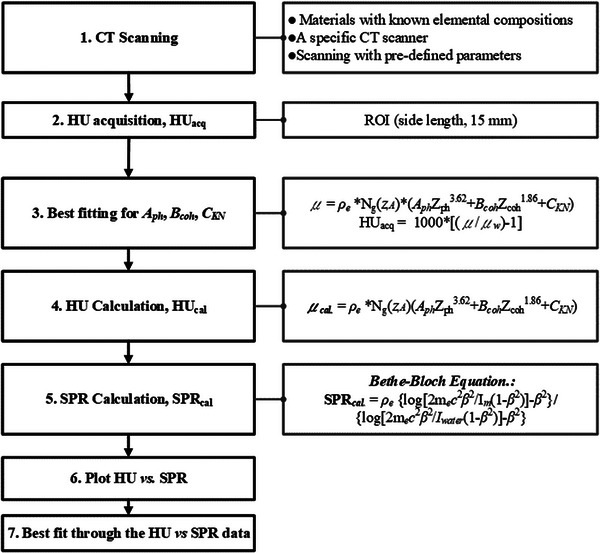
Workflow of the stoichiometric method, involving seven major steps. *μ* = linear attenuation coefficient; *ρ*_e_ = electron density; *Z* = atomic number; *Z_i_* = effective atomic number of the *i*th material; *A*_ph_, *B*_coh_, and *C*_KN_ = coefficients of photoelectric interaction, coherent scatter, and Compton scatter, respectively; *N*_g_ = number of electrons per volume; *m*_e_ *c* ^2^ = rest mass energy of an electron; *β* = proton speed relative to the speed of light; *I* = mean excitation energy of a material (m) and water (w).

In this study, a stoichiometric technique based on single‐energy CT (SECT)^16^, ^21^ was used. This technique has been extensively evaluated and used in particle therapy. Its purpose is to reduce the uncertainties introduced by the elemental compositions of artificial TEMs and human tissues by using a parameterized virtual CT scanner. In this study, seven steps were followed to perform this technique (Figure 1). First, a phantom with a known elemental composition was used to create the parameterized virtual CT scanner. An advanced electron density phantom (Model 1467; Sun Nuclear) consisting of a small cylinder (length = 16.5 cm, Φ = 20 cm), a large external sleeve (length = 16.5 cm, Φ = 30 × 40 cm^2^), and TEM rods (Table 1) was used to generate a curve. A small phantom and an assembled phantom were then designed to simulate the head and body, respectively. To mitigate the effect of beam hardening on the HU and SPR accuracy, each TEM rod was placed at the center of the head and body phantoms and individually scanned. Second, the averaged HU (HU_acq_) of each rod was then extracted from a square region of interest (ROI) with a side length of 15 mm at the central slice. The HU value for the curve generation was averaged from the data of the head and body phantoms to account for the influence of phantom size on the calculation of the SPR.^15^ Third, the coefficients of photoelectric interaction (*A*_ph_), coherent scatter (*B*_coh_), and Compton scatter (*C*_KN_) were determined for a parameterization equation based on the information (HU_acq_, RED, and effective atomic number) derived from the TEM rods. A total of 86 human tissues obtained from Woodard and While^22^ and ICRP‐report 23^23^were used to calculate the HU values (HU_cal_) and then plotted against the SPR values according to the fitted coefficients and Bethe‐Bloch equation,^24^ respectively. A fixed proton energy of 115 MeV was used to minimize the effect of single‐proton energy on SPR accuracy.^15^, ^16^ Linear fitting was then conducted separately for lung‐, soft‐tissue‐, and bone‐like groups. These groups were determined from the data published by Woodard and While.^22^ Finally, a piecewise HU‐to‐SPR conversion curve was generated by connecting the fitting lines between groups.^16^ In addition, conversion curves for FOV sizes of 50, 60, and 70 cm were generated. These curves were compared with the scanned data of all phantom setups to determine their effect on TEM rods.

**TABLE 1:** TEM rods used to generate HU‐to‐SPR conversion curves and the corresponding position indices of the heterogeneous phantom (Figure 4)

Tissue equivalent material	*ρ* (g/cm^3^)	*ρ* ^rel^_e_	Index
LN300 lung	0.29	0.28	13
LN450 lung	0.45	0.44	14
HE general adipose	0.96	0.94	5
HE breast 50/50	0.98	0.97	8
HE CT solid water	1.02	1.00	2, 3, 9, 11
True water	1.00	1.00	1
HE brain	1.05	1.02	6
HE liver	1.08	1.05	16
HE inner bone	1.21	1.16	12
CaCO3‐30% (bone)	1.33	1.27	10
CaCO3‐50% (bone)	1.56	1.46	4
HE cortical bone	1.93	1.78	7
HE Blood‐100 (B‐100)^a^	1.13	1.10	15

^a^ HE Blood‐100 (B‐100) was used only in the heterogeneous phantom study.

### 2.3 Homogeneous body phantom with an extension component

**FIGURE 2:**
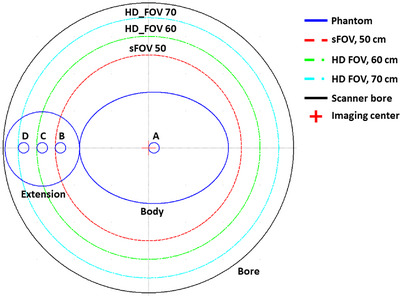
Body and extension phantom arrangement. Selected rods are placed at positions A to D and scanned individually to assess the Hounsfield unit (HU) and stopping power ratio (SPR) variations along the horizontal axis for different reconstructed field‐of‐views (FOVs). HD, high definition; sFOV, scanning field‐of‐view.

To investigate the HU and SPR variations at various off‐center distances (A to D) for the given FOV sizes, an assembled body phantom, and a customized extension phantom were used. A cylindrical extension (length = 10 cm, diameter = 20 cm) made of PMMA (density = 1.19 g/cm^3^) was fabricated. Figure 2 depicts the phantom arrangement. Selected TEM rods were placed at predefined positions and scanned individually. Lung (LN300), adipose, water, brain, inner bone, and cortical bone rods were then selected to cover materials from low to high densities for clinical applications. A setup involving a rod located at position A, 15 mm from the imaging center, and reconstructed with an FOV of 50 cm was used as the reference. Positions B, C, and D were located at the boundaries of FOVs with diameters of 50, 60, and 70 cm, respectively. The data acquired from positions C and D were used to examine the SPR variations in the eFOV region. A square ROI centered on the axis of each rod, with a side length of 15 mm at the central imaging slice, was used to obtain HU data for subsequent analyses.

### 2.4 Heterogeneous phantom

**FIGURE 3:**
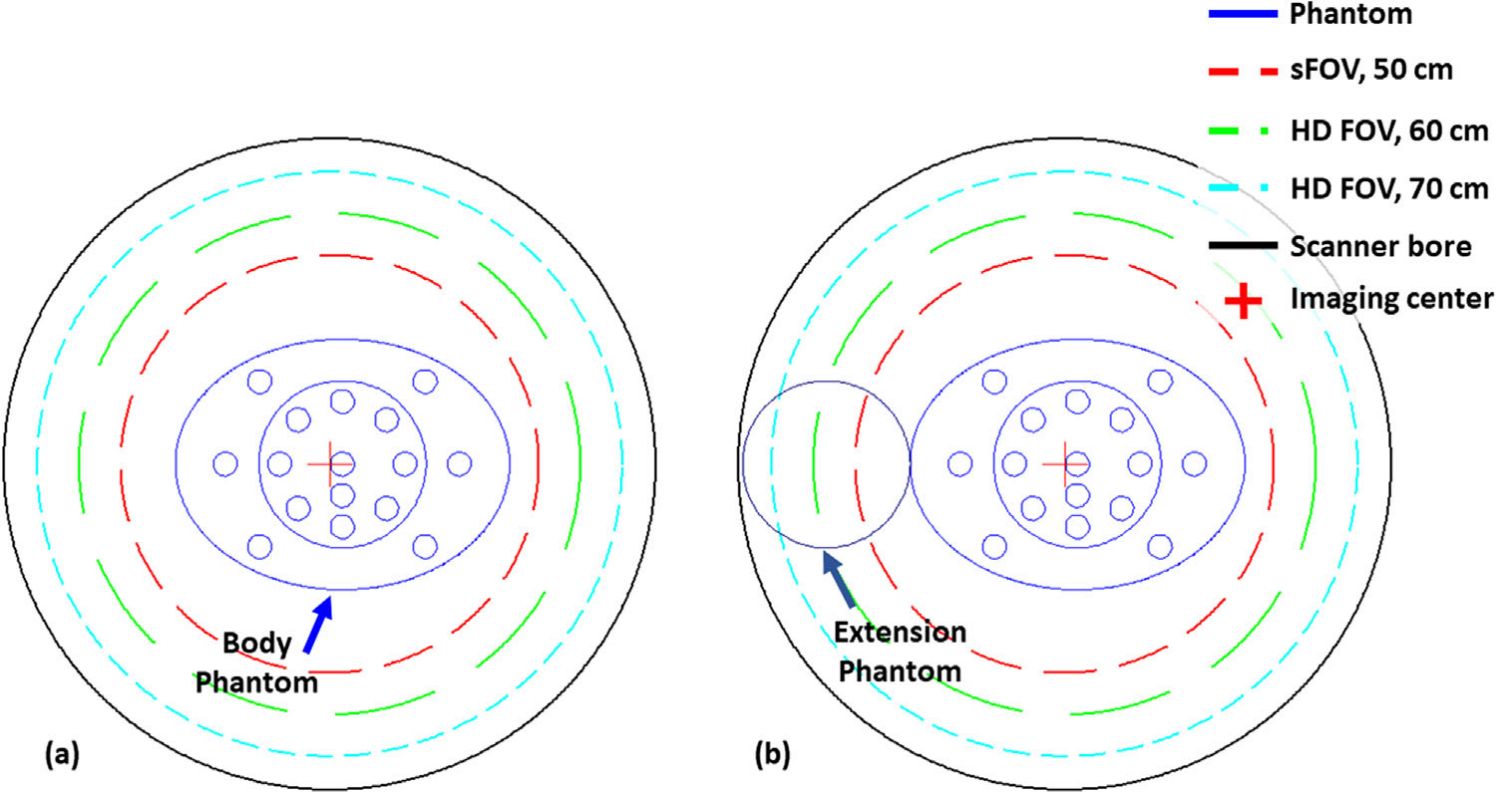
Tissue characterization phantom with 16 tissue‐equivalent material (TEM) rods scanned in the (a) absence and (b) presence of an extension phantom. FOV, field‐of‐view; HD, high definition; sFOV, scanning field‐of‐view.

**FIGURE 4:**
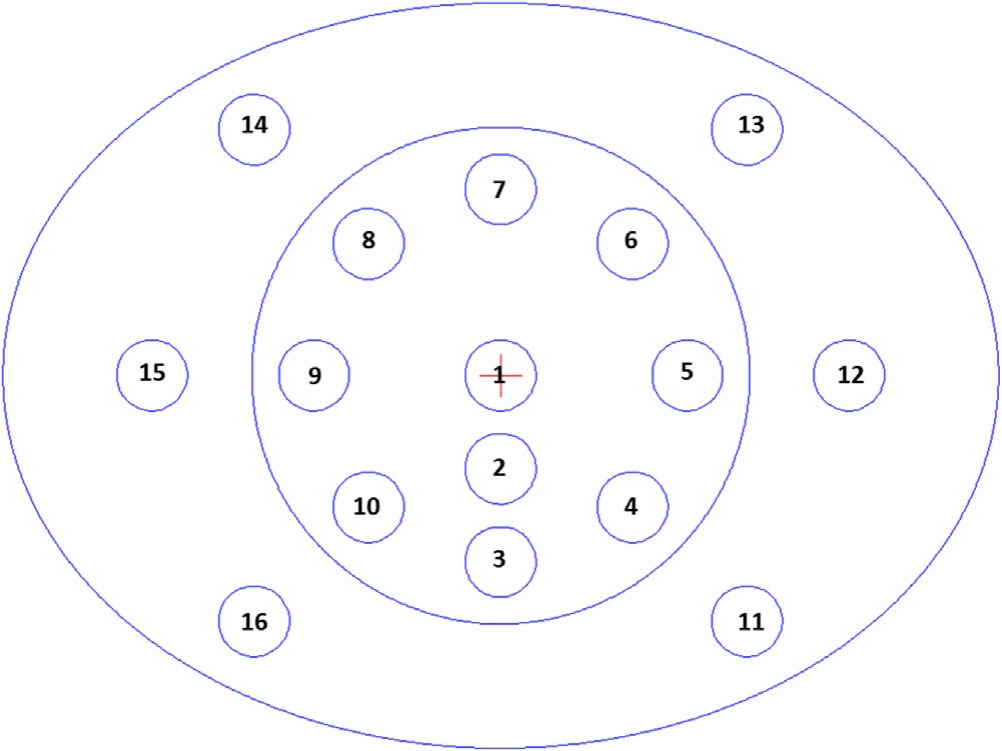
Position indices of tissue‐equivalent material (TEM) rod distribution used for measurement. A total of 16 rods were inserted into the body phantom and scanned simultaneously. The corresponding rod characteristics and position indices are listed in Table 1.

A body phantom with 16 TEM rods and an extension phantom were used to examine the effect of the eFOV approach, in the absence and presence of an extension phantom, on SPR estimation in a heterogeneous medium (Figure 3). To place the center of the body phantom as close as possible to the imaging center while accommodating the extension component, the body phantom was shifted 15 mm from the bore center. All rods were simultaneously scanned and reconstructed using various FOV sizes, including 50, 60, and 70 cm. The Gammex‐467‐like default rod distribution was applied (see Figure 4). Table 1 illustrates the relationship between the TEM rods and rod indices. In general, adequate rod distribution is crucial for mitigating the high‐ and low‐HU streaking artifacts resulting from the beam‐hardening effect (BHE), especially for low‐density materials (e.g., lung materials). In this study, the averaged value, including the HU and SPR values, was calculated from a square ROI with a side length of 15 mm centered on the axis of each rod at the central imaging slice. The image set generated from the conversion curve derived from the 50 cm FOV (CurveFOV50) and reconstructed using the 50 cm FOV (FOV50) was used as the reference configuration. Finally, the HU and SPR differences of various conversion curve – FOV combinations from the reference configuration were assessed.

## 3 RESULTS

### 3.1 HU‐to‐SPR conversion curve

**FIGURE 5:**
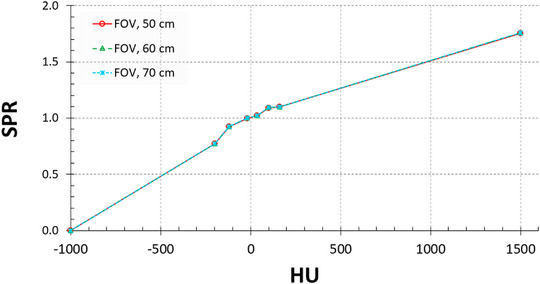
Conversion curves generated with a field‐of‐view (FOV) of 50 cm (sFOV) and with eFOVs of 60 and 70 cm. HU, Hounsfield unit; SPR, stopping power ratio.

Figure 5 depicts the HU‐to‐SPR conversion curves derived from the three aforementioned FOV diameters. The difference between these curves and the FOV50 curve was within 0.5% on average (data not shown). However, no discernible difference was observed between the curves. The three curves were applied to the image sets of a body phantom, for curve generation, and reconstructed using different FOV sizes.

### 3.2 Homogeneous body phantom with an extension component

**FIGURE 6:**
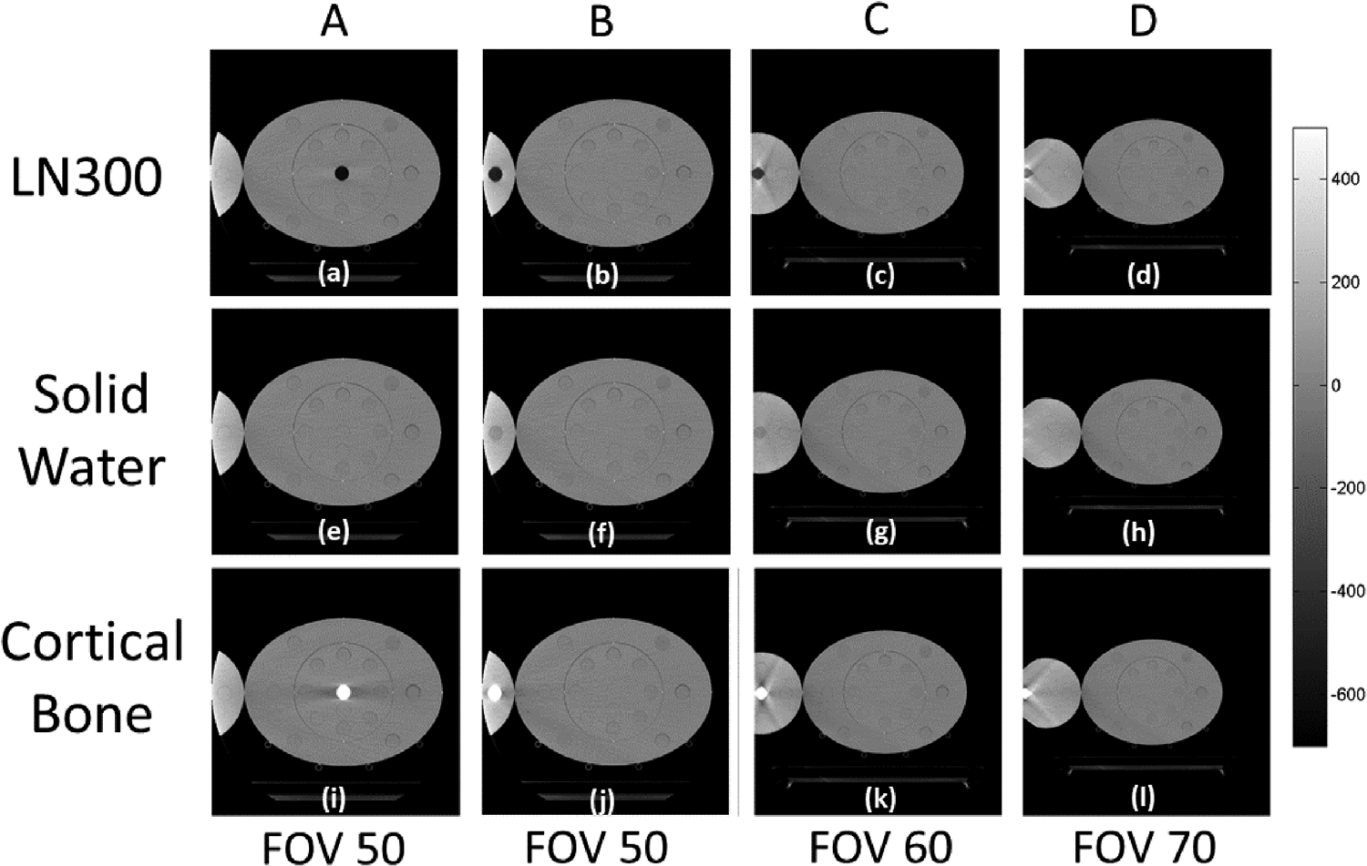
Reconstructed images of various field‐of‐view (FOV) sizes and rod positions (positions A to D) of lung (LN300, top row), solid water (middle row), and cortical bone (bottom row) tissue‐equivalent material (TEM) rods.

**FIGURE 7:**
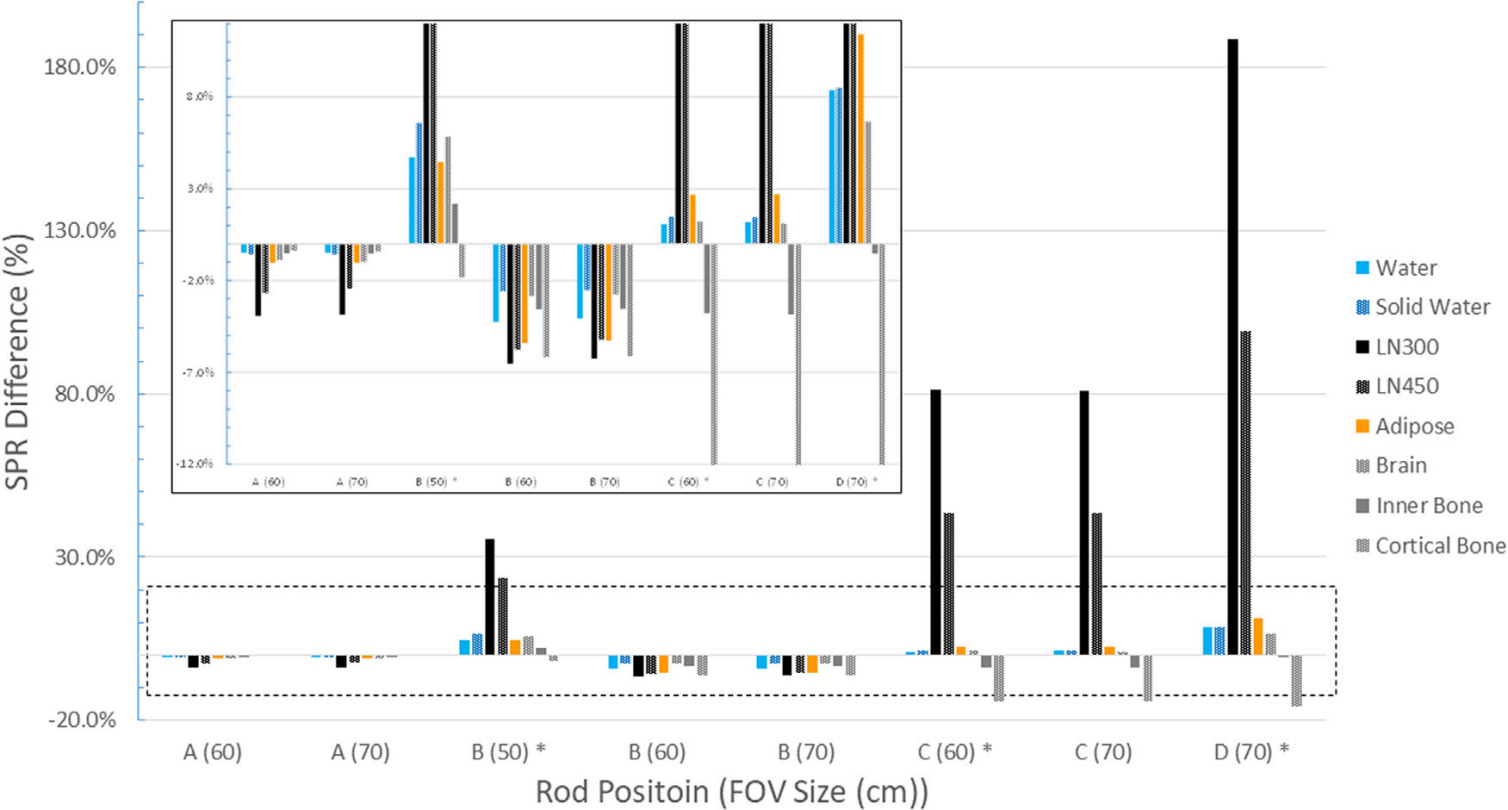
Stopping power ratio (SPR) differences at various phantom positions (positions A to D) for different field‐of‐view (FOV) sizes. The inset shows a zoomed‐in view of the area in the dashed rectangle. The rod position close to the FOV boundary is marked with an asterisk.

The results obtained from the various conversion curves were comparable. Only the results of the conversion curve derived from the 50 cm FOV (CurveFOV50) are shown. To provide a clear explanation, Figure 6 depicts only images demonstrating marked effects. Different from the images of solid water (Figure 6e–h), low‐ and high‐intensity streaking artifacts were obtained from the images of LN300 (Figure 6a–d) and cortical bone (Figure 6i–l) TEM rods. The level of artifact severity varied with material density and position relative to the FOV boundary. The high‐intensity streaks became severe when the rod was located close to the boundary of the FOV. Figure 7 depicts the SPR differences between the reference setup and the various combinations of rod positions and FOV sizes. When the rod was placed close to the imaging center (position A), the SPR values derived from the eFOV approach became systematically smaller than the reference configuration. Depending on the distance from the FOV boundary, the differences of the soft‐tissue, bone, and lung materials were 3.3% (1.8−11.4%), 4.8% (0.4−15.9%), and 39.6% (2.4−188.9%), respectively.

### 3.3 Heterogeneous phantom

**FIGURE 8:**
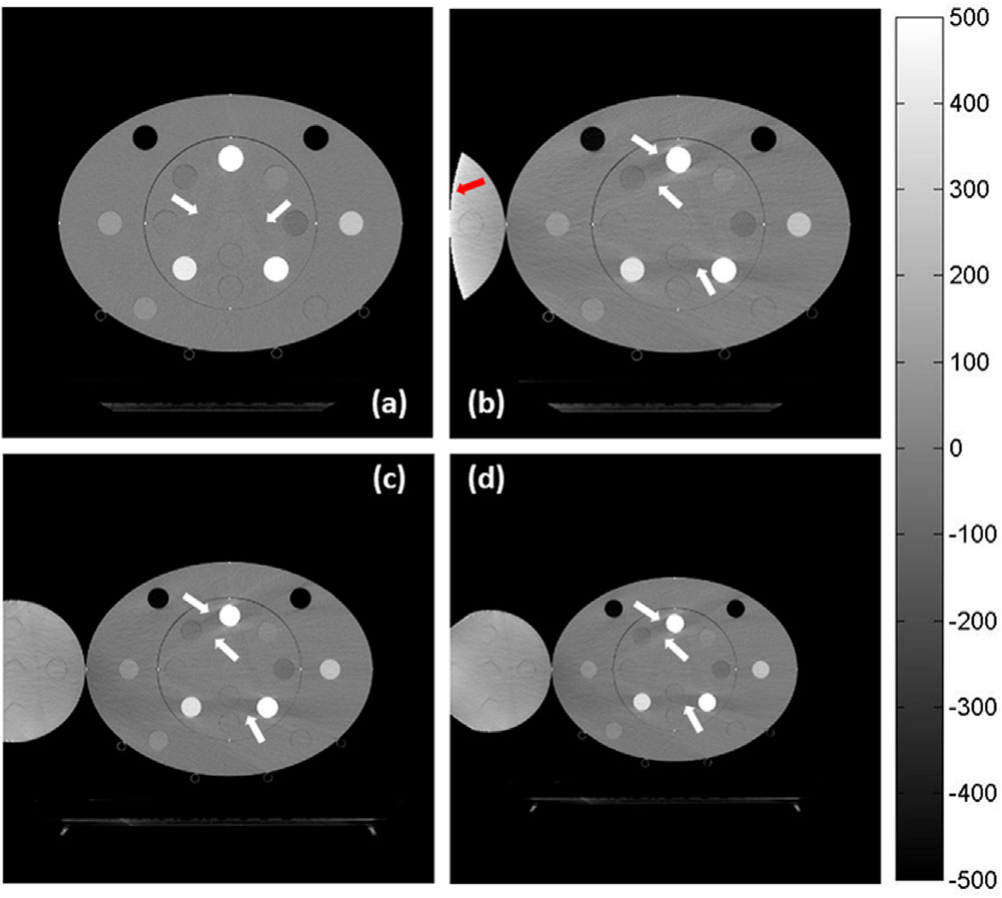
Reconstructed images in the absence of an extension phantom with a 50 cm FOV (sFOV) (a) and in the presence of an extension phantom with 50 (b), 60 (c), and 70 cm (d) FOVs. Both high‐ and low‐HU streak artifacts (white arrows) are observed in all images. A high‐intensity truncation artifact (red arrow) is also observed (b).

Figure 8 shows images in the absence (Figure 8a) and presence (Figure 8b–d) of an extension phantom. Because no discernible difference was observed between images reconstructed with different FOV sizes, in the absence of an extension phantom, only one image at the central slice reconstructed with a 50 cm FOV was observed. In subsequent analyses, an image reconstructed with a 50 cm FOV was considered as the reference image (Figure 8a,b). Figure 8c,d shows images reconstructed with 60 and 70 cm FOVs in the presence of an extension phantom. As shown in Figure 8a, only low‐HU streaking artifacts were observed between the three high‐density rods under the BHE. Because of the adequate rod distribution, these low‐HU streaks did not considerably affect the remaining TEM rods. However, as shown in Figure 8b–d, the low‐ and high‐HU streaking artifacts were severe in the presence of an extension phantom, resulting in a decrease in the accuracy of HU and SPR estimation. In addition, outside the sFOV region, the shape of the extension phantom became distorted, which agreed with earlier reports.^4^, ^13^

**FIGURE 9:**
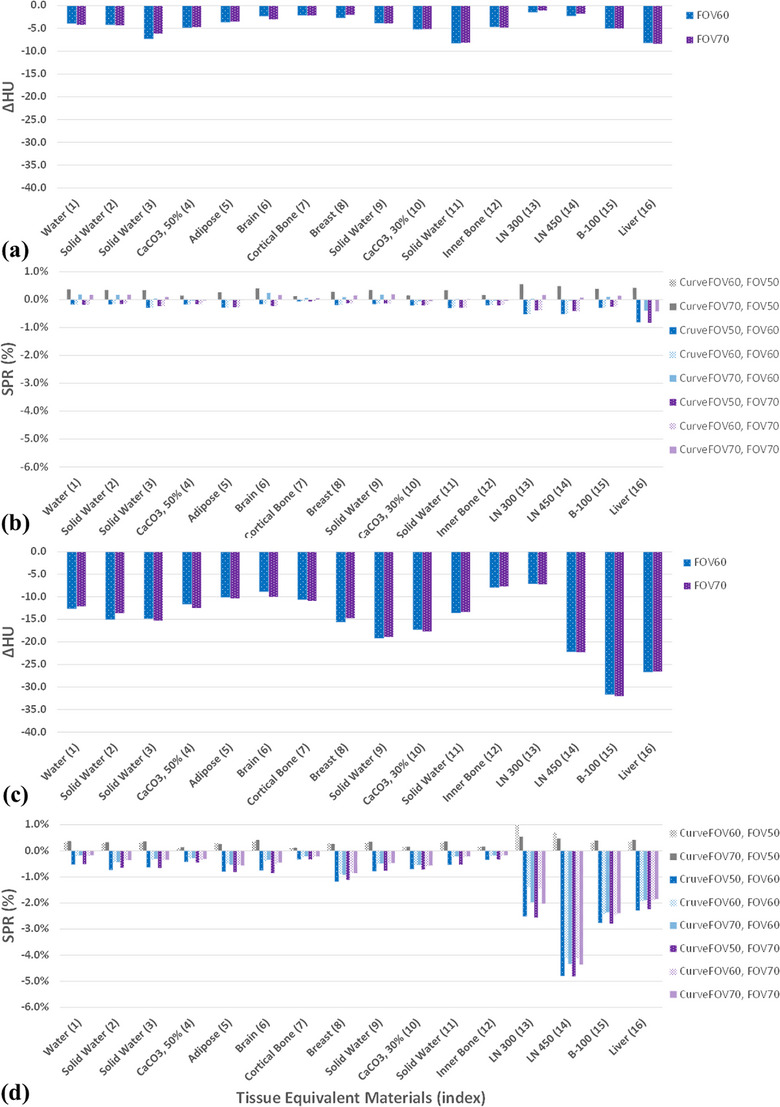
Averaged HU difference (ΔHU) and SPR difference (%) compared with the reference values in the absence (a, b) and presence (c, d) of an extension phantom.

Figure 9 depicts the HU and SPR variations of TEM rods in the absence and presence of an extension phantom compared with that reconstructed with a 50 cm FOV. In the absence of an extension phantom, the HU and SPR differences were on average 9 HU and 0.9%, respectively. Because of the extrapolation of the HU FOV approach for detruncation, the HU values of the eFOV approach were systematically smaller than the standard FOV value.^12^, ^20^ In addition, no substantial difference was observed between any of the combinations. However, in the presence of an extension phantom, the difference increased to 32 HU (Figure 9c) and 2.8% (Figure 9d), except for the lung materials. The lung materials were sensitive to high‐intensity artifacts, because of their low density and HU values. Therefore, they demonstrated the maximum SPR difference (4.8%). In addition, owing to the nature of the SECT‐based stoichiometric method, the trends of SPR and HU difference for the TEM rods were coherent, in general. Although the conversion curves generated with different field sizes resulted in slight SPR perturbations, the SPR difference was dominated by HU variations.

**FIGURE 10:**
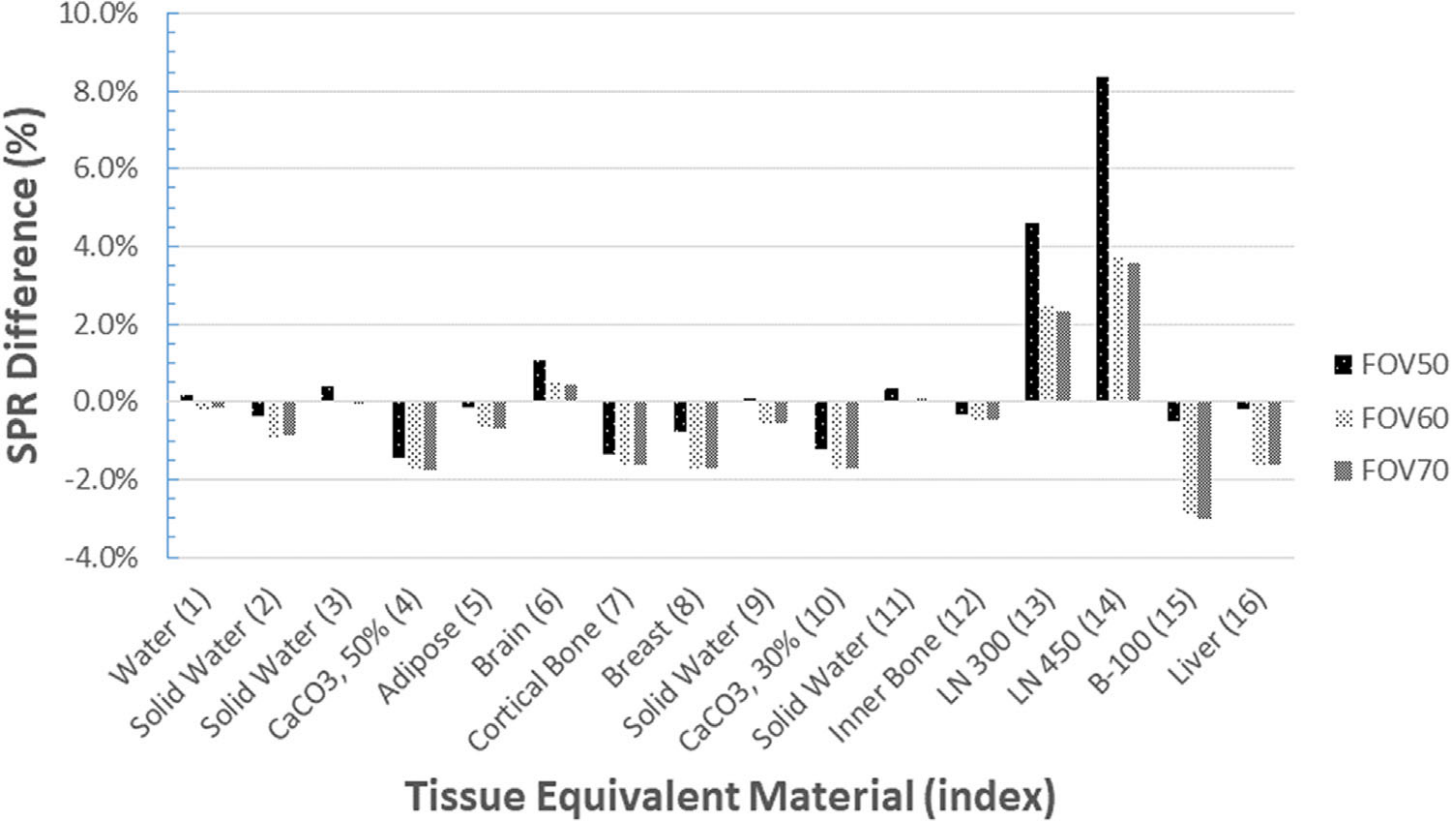
Stopping power ratio (SPR) differences for each FOV size between configurations in the absence and presence of an extension phantom. Only the results of the conversion curve generated from a 50 cm field‐of‐view (FOV) are plotted. Except for the low‐density lung materials, the difference was approximately 2%.

Figure 10 depicts the SPR difference of each FOV size between measurements in the absence and presence of an extension phantom. It also demonstrates the effect of the beam‐hardening artifacts caused by the extension phantom. However, only the results of CurveFOV50 are depicted. Depending on the location and material of TEM rods, the streaking artifacts resulted in an SPR difference of approximately 2%. Again, this difference considerably increased to approximately 8% for lung materials.

## 4 DISCUSSION

In IMPT applications, SPR data are routinely derived from HU values reconstructed using FOV sizes equivalent to sFOV values. However, given the increasing demand for CT simulators with wider dFOVs, the eFOV approach has been regarded as a potential alternative for CT simulators with limited sFOV sizes. In this study, the effects of the eFOV approach on the accuracy of SPR estimation were evaluated.

As shown in Figure 5, the conversion curves obtained with different FOV sizes were comparable. To generate detruncated images, extrapolation was conducted in the eFOV region between the sFOV and dFOV boundaries in both the projection and the image domains.^20^ In addition, a low‐pass Gaussian filter was used to generate a smooth outer object boundary. As a result, the eFOV approach did not alter the HU values inside the sFOV region, especially in the absence of image truncation, technically. To further minimize the effect of the eFOV approach on HU accuracy, TEM rods were placed at the center of the phantoms and sFOV in this study. Moreover, in the SECT‐based stoichiometric method, the HU values dominated the outcome of SPR estimation. Because the HU values obtained from the aforementioned FOV sizes were comparable, the conversion curves (Figure 5) and SPR values calculated from the curves were almost identical.

In the presence of an extension phantom, the HU and SPR estimation values of the extension approach were systematically lower than the reference values, except for those of lung materials (Figure 9). These results agree with those obtained in previous studies.^4^, ^12^ As indicated previously, the eFOV approach did not alter the data inside the sFOV region. However, when a smoothing filter was used at the object boundary, additional perturbation was introduced between the forward and backward projections. Consequently, the HU and SPR values inside the sFOV region changed. By contrast, in the absence of an extension phantom, the effect of the eFOV approach on SPR variation was limited.

As shown in Figures 6 and 8, clear high‐ and low‐intensity streaking artifacts were observed in the reconstructed images under the BHE. Generally, because the x‐ray sources of CT scanners emit polychromatic beams, low‐energy photons are more easily absorbed than high‐energy photons, thereby increasing the mean x‐ray energy. This phenomenon is referred to as the BHE, which may result in variations in the estimated HU and SPR values. Both phantom composition and size play an essential role in the strength of the BHE.^26^ The strength of the BHE corresponds to the phantom size, which is clearly observed in materials with extremely low and high densities because they substantially alter the spectrum of incident x‐ray beams. In this study, because low‐density lung rods were placed in a body phantom with an extension component, the SPR deviation was more pronounced than that of the other materials. Generally, linearization‐based BHCs are typically used in image reconstruction to mitigate the BHE for water and soft tissues. However, their efficacy is limited in the presence of strong streaking artifacts (e.g., those resulting from lung and bone materials). Therefore, to minimize the effect of streaking artifacts on image quality and quantitative analyses, iterative BHC (iBHC) methods have been proposed to account for different tissue types, especially bone.^27^, ^28^, ^29^ Chacko et al.^5^ evaluated the effectiveness of iBHC in improving the robustness of SPR estimation. They examined the effects of an iBHC method (Siemens Healthineers) on SPR and HU variations and compared them with those of stoichiometric methods based on SECT and dual‐energy CT (DECT). Their results indicated that the SPR deviation decreased from 2.6% to 1.4% and from 2.4% to 0.6% when the iBHC method and the SECT‐ and DECT‐based stoichiometric methods, respectively, were used. In that study, images were reconstructed with an FOV with a diameter of 50 cm. However, the relationship between the eFOV approach and the iBHC method has not been examined. Therefore, further research is required to determine the effect on SPR estimation.

Currently, a 3.5% (5%) uncertainty in HU‐to‐SPR conversion is commonly used in the majority of tissues (lungs) for margin design.^15^ In this study, the presence of an object outside the sFOV resulted in an SPR deviation of 2.8% for the majority of TEM rods and an SPR deviation of up to 4.8% for lung TEM rods. These results indicated a high SPR variation for proton applications. In addition, when rods were placed adjacent to the FOV boundary, high‐intensity artifacts were observed, especially in images reconstructed with a 50 cm FOV. Although these artifacts were acceptable for photons,^4^, ^14^ they resulted in an SPR deviation of more than 3% for the majority of tissues. Therefore, to increase the flexibility of beam arrangement, the target must be positioned as close as possible to the bore center, and the organs at risk (OARs) must be positioned as far as possible from the sFOV boundary.

## 5 CONCLUSIONS

This study investigated the effect of eFOV approaches on SPR estimation. The results indicated that the SPR variation increased from −1.1% to 4.8% for all TEM rods when an eFOV approach was used. Because of the synthetic effect in the BHE and the eFOV approach, the estimation of SPR variation was challenging. In addition, when the rods were placed close to the FOV boundary, strong artifacts were observed in the standard FOV size. Therefore, the target volumes and OARs must be placed as close as possible to the imaging center and as far as possible from the FOV boundary. The accuracy of SPR estimation has not been met the requirement using the eFOV approach, as a result, image reconstruction with a standard FOV size is recommended in IMPT applications.

## AUTHOR CONTRIBUTIONS

Chang‐Shiun Lin: Contributions to the conception, design of the work interpretation, and draft the work. Yi‐Chun Tsai: Contributions to interpretation, data acquisition, and revision. Liang‐Hsin Chen: Contributions to interpretation. Chun‐Wei Wang: Contributions to interpretation and draft the work. Chia‐Jung Wu: Contributions to data acquisition. Wan‐Yu Chen: Contributions to interpretation. Hsiang‐Kung Liang: Contributions to interpretation. Sung‐Hsin Kuo: Contributions to interpretation.

## CONFLICT OF INTEREST STATEMENT

The authors declare no conflicts of interest.
